# Predictors of Mortality in COVID-19 Patients in Southern California – Retrospective Multicenter Study

**DOI:** 10.7759/cureus.18137

**Published:** 2021-09-20

**Authors:** Chukwuemeka Umeh, Stella Maguwudze, Adrian Torbela, Shipra Saigal, Harpreet Kaur, Shadi Kazourra, Mahendra Aseri, Rakesh Gupta, Sumanta Chaudhuri, Rahul Gupta

**Affiliations:** 1 Internal Medicine, Hemet Global Medical Center, Hemet, USA; 2 Data Engineering & Business Intelligence, Hemet Global Medical Center, Hemet, USA; 3 Pulmonary and Critical Care Medicine, Hemet Global Medical Center, Hemet, USA

**Keywords:** intensive care unit (icu), creatine phosphokinase (cpk), lactate dehydrogenase (ldh), california, remdesivir, mortality, covid-19

## Abstract

Introduction

The majority of patients infected with coronavirus disease 2019 (COVID-19) recover from the illness after suffering mild to moderate symptoms, while approximately 20% progress to severe or critical disease, which may result in death. Understanding the predictors of severe disease and mortality in COVID-19 patients will help to risk stratify patients and improve clinical decision making. US data to inform this understanding are, however, scarce. We studied predictors of COVID-19 mortality in a cohort of 1,116 hospitalized patients in Southern California in the United States.

Methods

We conducted a retrospective cohort study of COVID-19 patients admitted at two hospitals in Southern California United States between March 2020 and March 2021. Bivariate and multivariate analyses of the relationship between mortality and other variables such as demographics, comorbidities, and laboratory values were performed, with a p-value of 0.05 considered as significant.

Results

The analysis involved 1,116 COVID-19 patients, of which 51.5% were males and 48.5% were females. Of the 1,116 patients, 81.6% were whites, 7.2% were blacks, and 11.2% were other races. After adjusting for co-variables, age (p<0.001), admission to intensive care unit (p< 0.001), use of remdesivir (p=0.018), C-reactive protein (CRP) levels (p<0.001), and lactate dehydrogenase (LDH) levels (p=0.039) were independently associated with mortality in our study. Gender, race, body mass index, presence of co-morbidities such as diabetes and hypertension, and use of steroid, statin, calcium channel blockers, angiotensin-converting enzyme inhibitors or angiotensin receptor blockers were not associated with mortality in the multivariate analysis.

Conclusion

In the cohort we studied, admission to intensive care unit was associated with decreased mortality while older age, use of remdesivir, and high levels of CRP and LDH were associated with increased mortality in COVID-19 patients.

## Introduction

Coronavirus disease 2019 (COVID-19) is caused by a novel coronavirus belonging to the family Coronaviridae and has caused significant morbidity and mortality globally [1]. The first cases were reported in China in December 2019 and a pandemic was declared on March 11, 2020, by the World Health Organization [1]. The infection mainly presents as fever, body aches, shortness of breath, malaise, and dry cough; some patients present with gastrointestinal symptoms such as diarrhea [1]. Most people infected with COVID-19 virus have mild disease and recover, but about 20% of patients go on to develop severe or critical disease that may result in death [2]. COVID-19 was first reported in the United States on January 21, 2020 [3]. Since then, there have been more than 32 million confirmed cases and over 500,000 COVID-19 deaths in the United States as of May 22, 2021. Greater than 10% of these deaths occurred in California [4].

Although prior COVID-19 studies on predictors of severe disease and mortality have been published from different locations globally, there is paucity of data from the United States. We now know from prior studies that advanced age, elevated inflammatory markers, presence of comorbidities such as diabetes and hypertension, and presence of ground glass opacities on imaging are associated with increased risk of mortality [5-8]. However, we do not clearly understand to what extent these factors predict death or survival in patient with COVID-19 in California considering the paucity of studies from the United States. Understanding the predictors of severe disease or mortality in COVID-19 patients will help to risk stratify patients and inform clinical decisions. To our knowledge, this is the first study in Southern California, which examines predictors of mortality in patients hospitalized with COVID-19.

## Materials and methods

This is a retrospective study of 1,116 COVID-19 patients who were admitted in two hospitals in Southern California in the United States between March 2020 and March 2021. The study includes all patients who sought care at the two facilities for COVID-19 and non-COVID-19-related symptoms and were diagnosed with COVID-19 through a positive polymerase chain reaction nasopharyngeal swab. For COVID-19 patients who were admitted in the hospital more than once during the study period for COVID-19 symptoms, their last hospital admission data were used for the study. We used COVID-19 patients' last hospital admission for COVID-19-related symptoms because some patients died from COVID-19-related complications during their subsequent hospital admission. Using the last hospital admission prevented a misclassification bias because we would otherwise have misclassified some patients who died as having survived if we only used their initial hospitalization for COVID-19 symptoms. Relevant deidentified data were extracted from the electronic medical record including patients age, sex, race, ethnicity, marital status, comorbidities, laboratory results on admission, date of admission, date of discharge, medications they received while on admission, and disposition at discharge. The decision on each patient’s level of care and what treatment to start on each patient was done by the hospitals' COVID-19 team consisting of a pulmonary and critical care specialist and hospitalists, in consultation with an infectious disease specialist and pharmacist. Every COVID-19 patient was seen by one of the members of the COVID-19 team.

Univariate analysis of study variables was done using means and percentages. Bivariate analysis of the relationship of different study variables with mortality was done using chi-square and t-test, with a p-value of 0.05 considered as significant. Finally, backward selection cox regression analysis was done using mortality as a dependent variable. All other variables such as patients' age, sex, race, ethnicity, marital status, comorbidities, medication that patients received while in the hospital, and laboratory results on admission were included as independent variables. The effect was expressed in terms of hazards ratio with 95% confidence interval. Statistical analysis was done using IBM SPSS version 27 (IBM Corp, Armonk, NY). The study was approved by WIRB-Copernicus Group institutional review board (IRB) and the study IRB approval number is 13410516.

## Results

Baseline study characteristics

The analysis involved 1,116 COVID-19 patients, of which 51.5% were males and 48.5% females. Of the 1,116 patients, 81.6% were whites, 7.2% were blacks, and 11.2% were other races. Nineteen percent of the patients were placed on a ventilator during their hospital admission and 26.4% of the patients died during hospital admission. 

Bivariate analysis

In the bivariate analysis of categorical variables, male sex (p<0.001), ventilator dependence (p<0.001), admission to intensive care unit (ICU) (p<0.001), presence of hypertension (p=0.009), chronic kidney disease (p<0.001), acute kidney injury (p<0.001), congestive heart failure (p<0.001), chronic obstructive pulmonary disease (p=0.005), coronary artery disease (p<0.001), beta blockers use (p=0.015), calcium channel blocker use (p=0.01), and steroid use (p<0.001) were associated with increased mortality (Table 1). Patients’ race, diabetes, cerebrovascular accident, and use of angiotensin-converting-enzyme inhibitors (ACEi) or angiotensin receptor blockers (ARBs) were not associated with increased mortality (Table 1).

**Table 1:** Bivariate analysis of the relationship between categorical variables and mortality ACEi: angiotensin-converting enzyme inhibitors; ARB: angiotensin receptor blockers

Variable	Expired	Survived	p-Value
Gender			
Male	180 (31.3%)	395 (68.7%)	<0.001
Female	115 (21.3%)	426 (78.7%)	
Race			
White	239 (26.2%)	672 (73.8%)	0.156
Black	16 (20%)	64 (80%)	
Others	40 (32%)	85 (68%)	
Ventilator use			
Yes	169 (81.6%)	38 (18.4%)	<0.001
No	126 (13.9%)	783 (86.1%)	
Intensive care unit admission			
Yes	195 (81.9%)	43 (18.1%)	<0.001
No	100 (11.4%)	778 (88.6%)	
Diabetes			
Yes	143 (28.7%)	356 (71.3%)	0.13
No	152 (24.6%)	465 (75.4%)	
Hypertension			
Yes	197 (29.2%)	477 (70.8%)	0.009
No	98 (22.2%)	344 (77.8%)	
Chronic kidney disease			
Yes	80 (35.2%)	147 (64.8%)	<0.001
No	215 (24.2%)	674 (75.8%)	
Acute kidney injury			
Yes	137 (46.6%)	157 (53.4%)	<0.001
No	158 (19.2%)	664 (80.8%)	
Congestive heart failure			
Yes	73 (37.6%)	121 (62.4%)	<0.001
No	222 (24.1%)	700 (75.9%)	
Chronic obstructive pulmonary disease			
Yes	56 (35.7%)	101 (64.3%)	0.005
No	239 (24.9%)	720 (75.1%)	
Cerebrovascular accident (stroke)			
Yes	25 (34.2%)	48 (65.8%)	0.117
No	270 (25.9%)	773 (74.1%)	
Coronary artery disease			
Yes	76 (36.9%)	130 (73.6%)	<0.001
No	219 (24.1%)	691 (75.9%)	
Use of ACEi or ARBs			
Yes	64 (22.9%)	216 (77.1%)	0.117
No	231 (27.6%)	605 (72.4%)	
Use of beta-blocker			
Yes	108 (31.2%)	238 (68.8%)	0.015
No	187 (24.3%)	583 (75.7%)	
Use of calcium channel blocker			
Yes	89 (32.4%)	186 (67.6%)	0.01
No	206 (24.5%)	635 (75.5%)	
Use of statin			
Yes	140 (28.2%)	357 (71.8%)	0.239
No	155 (25%)	464 (75%)	
Use of steroid			
Yes	239 (32.5%)	496 (67.5%)	<0.001
No	56 (14.7%)	325 (85.3%)	

In the bivariate analysis of the continuous variables, longer duration of hospital stay (p<0.001), older age (p<0.001), and higher admission C-reactive protein (CRP) (p<0.001), lactate dehydrogenase (LDH) (p<0.001), d-dimer (p<0.001), ferritin (p=0.04), troponin (p<0.001), platelet count (p<0.001), white blood cell count (p<0.001), potassium (p<0.001), and creatinine (<0.001) were associated with increased mortality (Table 2). Higher body mass index (BMI) (p=0.75) was not associated with increased mortality (Table 2).

**Table 2:** Bivariate analysis of the relationship between continuous variables and mortality

	Expired	Number of patients	Mean	Standard deviation	p-Value
Length of stay (days)	No	821	7.72	7.21	<0.001
	Yes	295	12.66	9.57	
Age (years)	No	821	63.48	18.31	<0.001
	Yes	295	71.20	13.53	
Body mass index	No	821	30.84	8.85	0.75
	Yes	295	30.65	9.06	
C-reactive protein	No	636	8.00	6.22	<0.001
	Yes	249	11.63	6.43	
Lactate dehydrogenase	No	626	339.05	433.13	<0.001
	Yes	238	581.04	626.22	
D-dimer	No	691	1098.00	1319.16	<0.001
	Yes	257	1954.26	1612.68	
Ferritin	No	470	662.14	2430.87	0.04
	Yes	211	1107.52	2973.51	
Troponin	No	747	0.13	0.53	<0.001
	Yes	277	0.43	1.69	
Creatine phosphokinase	No	557	254.89	736.69	<0.001
	Yes	217	850.33	3985.84	
Platelet	No	812	254.36	114.64	<0.001
	Yes	278	221.23	102.39	
White blood cell	No	819	9.10	5.16	<0.001
	Yes	281	12.32	6.65	
Potassium	No	814	4.18	0.63	<0.001
	Yes	280	4.49	0.82	
Creatinine	No	814	1.42	1.99	<0.001
	Yes	280	2.13	2.38	

Multivariate analysis

After adjusting for co-variables including patients’ demographics, comorbidities, laboratory results on admission and other medications that patients received while hospitalized, age (p<0.001), admission to ICU (p< 0.001), CRP (p<0.001), LDH (p=0.039), and use of remdesivir (0.018) were independently associated with mortality in COVID-19 patients in our study (Table 3).

**Table 3:** Multivariate cox regression (proportional hazard) analysis of factors that affect mortality in COVID-19 patients CI: confidence interval; COVID-19: coronavirus disease 2019

	Regression coefficient	Standard error	p-Value	Hazard ratio	95% CI for hazard ratio	
					Lower	Upper
Intensive care unit admission	-1.059	0.159	0.000	0.347	0.254	0.474
C-reactive protein	0.042	0.011	0.000	1.043	1.021	1.066
Lactate dehydrogenase	0.000	0.000	0.039	1.000	1.000	1.000
Remdesivir use	0.337	0.143	0.018	1.401	1.059	1.853
Age	0.034	0.006	0.000	1.034	1.023	1.046

A one-year increase in age was associated with 1.03 increased risk of mortality. Admission to the ICU resulted in 0.35 decreased risk of mortality. A one-unit increase in CRP was associated with 1.04 increased risk of mortality and one-unit increase in LDH is associated with 1.0 increased risk of mortality.

Gender, race, BMI, presence of co-morbidities such as diabetes and hypertension, and use of steroids, beta-blockers, ACEi, or ARBs were not associated with mortality in the multivariate analysis (Table 3).

## Discussion

Our analysis of mortality predictors in COVID-19 patients admitted in two hospitals in Southern California found multiple independent predictors of mortality. While some of our findings were consistent with findings in previous studies, other findings were unexpected. Consistent with prior studies, increased age appears to be a strong risk factor for COVID-19 mortality in our study. A one-year increase in age was associated with 1.03 increased risk of mortality after adjusting for other co-variables. Age has consistently been found to be an important independent risk factor for both severe disease and mortality in COVID-19 patients and mortality increases with age, with the highest mortality reported among people over 80 years of age [2,9-12].

Our study found that being admitted in ICU resulted in 0.35 decreased risk of mortality. In our study, 82% of patients who were admitted to the ICU died and we had expected that ICU admission had no effect on mortality because of the high mortality rate of those admitted to ICU. However, contrary to our expectation, being admitted to the ICU significantly increased the chances of survival. In some health facilities, including the facilities in our study, due to pandemic pressures on limited ICU services, there was widespread use of advanced respiratory support (non-invasive ventilation or high-flow nasal oxygen) outside ICUs and ICU admission was reserved for patients that were deemed to be more severe [13]. However, based on the result of this study, severe or critically ill COVID-19 patients who were not admitted to the ICU, including patients who requested not to be resuscitated or intubated, were more likely to die compared to those that were admitted to ICU. Patients who requested not to be resuscitated or intubated were usually not admitted to the ICU irrespective of their sickness severity and this might explain the mortality benefit with ICU admission. Unfortunately, we did not have data on the number of COVID-19 patients who requested not to be resuscitated or intubated and could not adjust for these in our regression model. Conversely, close monitoring and early intervention for patients in ICU could explain the mortality benefit seen with ICU admission. However, our study did not show any mortality benefit with ventilator use.

Furthermore, we found that the use of remdesivir was associated with increased risk of mortality after adjusting for patients’ demographics, comorbidities, laboratory results on admission, and other medications that patients received while hospitalized. Though remdesivir has been found to be beneficial when used early in the course of COVID-19 illness, studies have shown that, similar to our finding, it may not be beneficial in the later stage of COVID-19 illness or in critically ill patients requiring mechanical ventilation [14-17]. This is because while remdesivir may be beneficial in the initial phase of viral replication in COVID-19 patients, severe disease in Covid-19 patient is mainly caused by the host's aggressive inflammatory response against COVID-19 virus and remdesivir might not be very beneficial at that stage [18,19]. The reason for the increased mortality with remdesivir in our study is not clear but one possible explanation is that the increased mortality could be secondary to remdesivir-associated bradycardia. The use of remdesivir has been associated with bradycardia in COVID-19 patients and bradycardia has been associated with increased mortality in COVID-19 patients [20-22]. 

The use of corticosteroids including dexamethasone or methylprednisolone was not associated with decreased mortality in our study after adjusting for patients’ demographics, comorbidities, laboratory results on admission, and other medications that patients received while hospitalized. Prior studies have shown that use of steroids lowers mortality among severe or critically ill patients who were and were not receiving invasive mechanical ventilation, irrespective of patients' age or gender by modulating inflammation-mediated lung injury and thereby reducing progression to respiratory failure and death [23,24]. Both high- and low-dose steroid use has been associated with decreased mortality, and, currently, there was no evidence suggesting that a higher dose of steroid is associated with significant greater benefit than a lower dose [23,25]. In the landmark RECOVERY trial that showed that dexamethasone has mortality benefit in COVID-19 patients, less than 0.1% of patients received concomitant remdesivir and the study did not report the proportions of patients who were on high-flow device or non-invasive ventilation or the amount of oxygen that patients received [24]. In our study, 60% of the patients who received steroid also received remdesivir and the lack of mortality benefit with steroid seen in our study may be due to the difference between our study population and the studies that showed mortality benefit with steroids.

We found that high CRP and LDH levels were associated with increased risk of mortality. A one-unit increase in CRP was associated with 1.04 increased risk of mortality and one-unit increase in LDH was associated with 1.0 increased risk of mortality. LDH and CRP are markers of acute inflammation and hypoxia in COVID-19 patients and increased levels of CRP and LDH correlate with increased severity of illness and increased risk of death [26-28].

Our study has some limitations. First, our study is a retrospective observational study and though we adjusted for major comorbidities and confounders in our analysis (Tables 1, 2), we may not have adjusted for some unmeasured or unknown confounders. However, we did not have data on the number of COVID-19 patients who requested not to be resuscitated or intubated and those who received COVID-19 vaccine and could not adjust for these in our regression model. Secondly, patients’ medications included in the analysis were based on information in the hospital electronic medical record which may not capture all the medication patients were taking outpatient and could have led to misclassification bias; however, we believe that the chance of this happening is minimal and will not have substantially affected the result of our study. Thirdly, the findings of our study are only generalizable to hospitalized COVID-19 population.

## Conclusions

In conclusion, this is a retrospective study of 1,116 COVID-19 patients who were admitted at two hospitals in Southern California, United States, between March 2020 and March 2021. After adjusting for independent variables, older age, high levels of CRP and LDH, and use of remdesivir were associated with increased mortality in our study. Conversely, admission to ICU was associated with decreased mortality while there was no mortality benefit with the use of steroid, statin, calcium channel blockers, or ACEi and ARB. Increased BMI, hypertension, and diabetes were not independently associated with mortality. There is need for more research on the mortality benefit of ICU admission for COVID-19 patients who request not to be resuscitated or intubated. There is also need for more studies on the mortality benefit of concurrent use of remdesivir and steroids in COVID-19 patients.

## References

[1] Parasher A (2021). COVID-19: current understanding of its pathophysiology, clinical presentation and treatment. Postgrad Med J.

[2] (2021). World Health Organization. Report of the WHO-China Joint Mission on Coronavirus Disease 2019 (COVID-19). https://www.who.int/docs/default-source/coronaviruse/who-china-joint-mission-on-covid-19-final-report.pdf.

[3] (2021). CDC. First Travel-related Case of 2019 Novel Coronavirus Detected in United States. https://www.cdc.gov/media/releases/2020/p0121-novel-coronavirus-travel-case.html..

[4] (2021). CDC. United States COVID-19 Cases, Deaths, and Laboratory Testing (NAATs) by State, Territory, and Jurisdiction. https://covid.cdc.gov/covid-data-tracker/.

[5] Tian W, Jiang W, Yao J (2020). Predictors of mortality in hospitalized COVID-19 patients: a systematic review and meta-analysis. J Med Virol.

[6] Mesas AE, Cavero-Redondo I, Álvarez-Bueno C, Sarriá Cabrera MA, Maffei de Andrade S, Sequí-Dominguez I, Martínez-Vizcaíno V (2020). Predictors of in-hospital COVID-19 mortality: a comprehensive systematic review and meta-analysis exploring differences by age, sex and health conditions. PLoS One.

[7] Imam Z, Odish F, Gill I (2020). Older age and comorbidity are independent mortality predictors in a large cohort of 1305 COVID-19 patients in Michigan, United States. J Intern Med.

[8] Aly MH, Rahman SS, Ahmed WA, Alghamedi MH, Al Shehri AA, Alkalkami AM, Hassan MH (2020). Indicators of critical illness and predictors of mortality in COVID-19 patients. Infect Drug Resist.

[9] Romero Starke K, Petereit-Haack G, Schubert M, Kämpf D, Schliebner A, Hegewald J, Seidler A (2020). The age-related risk of severe outcomes due to COVID-19 infection: a rapid review, meta-analysis, and meta-regression. Int J Environ Res Public Health.

[10] Chen T, Dai Z, Mo P (2020). Clinical characteristics and outcomes of older patients with coronavirus disease 2019 (COVID-19) in Wuhan, China: a single-centered, retrospective study. J Gerontol A Biol Sci Med Sci.

[11] Posso M, Comas M, Román M (2020). Comorbidities and mortality in patients with COVID-19 aged 60 years and older in a university hospital in Spain. Arch Bronconeumol (Engl Ed).

[12] O'Driscoll M, Ribeiro Dos Santos G, Wang L (2021). Age-specific mortality and immunity patterns of SARS-CoV-2. Nature.

[13] Armstrong RA, Kane AD, Cook TM (2020). Outcomes from intensive care in patients with COVID-19: a systematic review and meta-analysis of observational studies. Anaesthesia.

[14] Young B, Tan TT, Leo YS (2021). The place for remdesivir in COVID-19 treatment. Lancet Infect Dis.

[15] Beigel JH, Tomashek KM, Dodd LE (2020). Remdesivir for the treatment of Covid-19 - final report. N Engl J Med.

[16] Garibaldi BT, Wang K, Robinson ML (2021). Comparison of time to clinical improvement with vs without remdesivir treatment in hospitalized patients with COVID-19. JAMA Netw Open.

[17] Wang Y, Zhang D, Du G (2020). Remdesivir in adults with severe COVID-19: a randomised, double-blind, placebo-controlled, multicentre trial. Lancet.

[18] Tay MZ, Poh CM, Rénia L, MacAry PA, Ng LF (2020). The trinity of COVID-19: immunity, inflammation and intervention. Nat Rev Immunol.

[19] Ruan Q, Yang K, Wang W, Jiang L, Song J (2020). Clinical predictors of mortality due to COVID-19 based on an analysis of data of 150 patients from Wuhan, China. Intensive Care Med.

[20] Gubitosa JC, Kakar P, Gerula C (2020). Marked sinus bradycardia associated with remdesivir in COVID-19: a case and literature review. JACC Case Rep.

[21] Touafchia A, Bagheri H, Carrié D, Durrieu G, Sommet A, Chouchana L, Montastruc F (2021). Serious bradycardia and remdesivir for coronavirus 2019 (COVID-19): a new safety concerns. Clin Microbiol Infect.

[22] Kumar S, Arcuri C, Chaudhuri S, Gupta R, Aseri M, Barve P, Shah S (2021). A novel study on SARS-COV-2 virus associated bradycardia as a predictor of mortality-retrospective multicenter analysis. Clin Cardiol.

[23] Sterne JA, Murthy S, Diaz JV (2020). Association between administration of systemic corticosteroids and mortality among critically ill patients with COVID-19: a meta-analysis. JAMA.

[24] Horby P, Lim WS, Emberson JR (2021). Dexamethasone in hospitalized patients with Covid-19. N Engl J Med.

[25] Piccica M, Lagi F, Trotta M, Spinicci M, Zammarchi L, Bartoloni A; COCORA Working Group (2021). High-dose steroids for the treatment of severe COVID-19. Intern Emerg Med.

[26] Li C, Ye J, Chen Q (2020). Elevated lactate dehydrogenase (LDH) level as an independent risk factor for the severity and mortality of COVID-19. Aging (Albany NY).

[27] Aloisio E, Chibireva M, Serafini L, Pasqualetti S, Falvella FS, Dolci A, Panteghini M (2020). A comprehensive appraisal of laboratory biochemistry tests as major predictors of COVID-19 severity. Arch Pathol Lab Med.

[28] Huang I, Pranata R, Lim MA, Oehadian A, Alisjahbana B (2020). C-reactive protein, procalcitonin, D-dimer, and ferritin in severe coronavirus disease-2019: a meta-analysis. Ther Adv Respir Dis.

